# Endotoxin-Induced Inflammation Suppresses the Effect of Melatonin on the Release of LH from the Ovine *Pars Tuberalis* Explants—Ex Vivo Study

**DOI:** 10.3390/molecules22111933

**Published:** 2017-11-10

**Authors:** Karolina Wojtulewicz, Dorota Tomaszewska-Zaremba, Andrzej Przemysław Herman

**Affiliations:** The Kielanowski Institute of Animal Physiology and Nutrition, Polish Academy of Sciences, Instytucka 3 Street, 05-110 Jabłonna, Poland; k.wojtulewicz@ifzz.pl (K.W.); d.tomaszewska@ifzz.pl (D.T.-Z.)

**Keywords:** melatonin, lipopolysaccharide, luteinizing hormone, GnRH, melatonin receptors, *Pars Tuberalis*

## Abstract

The secretion of the hormone melatonin reliably reflects environmental light conditions. Among numerous actions, in seasonal breeders, melatonin may regulate the secretion of the gonadotropins acting via its corresponding receptors occurring in the *Pars Tuberalis* (*PT*). However, it was previously found that the secretory activity of the pituitary may be dependent on the immune status of the animal. Therefore, this study was designed to determine the role of melatonin in the modulation of luteinizing hormone (LH) secretion from the *PT* explants collected from saline- and endotoxin-treated ewes in the follicular phase of the oestrous cycle. Twelve Blackhead ewes were sacrificed 3 h after injection with lipopolysaccharide (LPS; 400 ng/kg) or saline, and the *PTs* were collected. Each *PT* was cut into 4 explants, which were then divided into 4 groups: I, incubated with ‘pure’ medium 199; II, treated with gonadotropin-releasing hormone (GnRH) (100 pg/mL); III, treated with melatonin (10 nmol/mL); and IV, incubated with GnRH and melatonin. Melatonin reduced (*p* < 0.05) GnRH-induced secretion of LH only in the *PT* from saline-treated ewes. Explants collected from LPS-treated ewes were characterized by lower (*p* < 0.05) GnRH-dependent response in LH release. It was also found that inflammation reduced the gene expression of the GnRH receptor and the MT1 melatonin receptors in the *PT*. Therefore, it was shown that inflammation affects the melatonin action on LH secretion from the *PT*, which may be one of the mechanisms via which immune/inflammatory challenges disturb reproduction processes in animals.

## 1. Introduction

Melatonin (*N*-acetyl-5-methoxytryptamine) is a hormone synthesized by pinealocytes in the pineal gland. Its secretion begins after darkness and rapidly stops after sunrise [1]. In sheep, nocturnal concentration of melatonin and the duration of its release depend on the photoperiodic conditions. Melatonin is secreted in the highest concentrations and for the longest period in the winter during the short-day photoperiod, whereas the lowest concentrations and the shortest duration of melatonin are found in the summer during the long-day photoperiod. The melatonin signal is considered to be involved in the regulation of reproduction [2]. In sheep, the duration of elevated melatonin levels over the long spring–summer days provides the signal to synchronize the circannual rhythm of reproductive neuroendocrine activity. The shortening days between the summer solstice and the autumn equinox are the critical signal involved in timing the end of reproductive activity in mid-winter, which contributes to ensuring the proper duration of the breeding season [3].

It is considered that melatonin at the hypothalamic level may influence reproduction by affecting gonadotropin-releasing hormone (GnRH) secretion. In a study on ovariectomized ewes with micro-implants containing melatonin placed in the medial-basal hypothalamus, increased release of luteinizing hormone (LH) was found [4]. However, melatonin receptors were either not found in the sheep hypothalamus or were detected in small numbers in the hypothalamic structures involved in the regulation of reproductive processes [5]. Therefore, the melatonin implant placed in the preoptic area failed to influence GnRH/LH secretion [6]. Melatonin receptors (MT) were found to be expressed in the premammillary hypothalamus, and melatonin delivered into this area stimulated LH secretion [7]. However, it is postulated that melatonin may modulate gonadotropin synthesis directly at the level of the pituitary gland reaching the discrete region called the *Pars Tuberalis (PT)* [8]. The *PT* is an exclusive pituitary structure in close anatomical contact with the medial-basal hypothalamus, median eminence (ME), and the third ventricle of the brain [9]. The *PT* is composed of follicular cells, *PT*-specific secretory cells, and *Pars Distalis (PD)*-like cells [10]. Gonadotrophs from the *PT* contain and secrete some amount of LH and follicle-stimulating hormone (FSH) [10]. Therefore, it is suggested that the *PT* supports the secretory function of the *PD* [11]. It is thought that the *PT* may mediate the effect of melatonin on neuroendocrine function and may be involved in the photoperiodic regulation of pituitary hormone secretion, because the expression of melatonin receptors is found exclusively in the *PT*, while it is absent in the *PD* [8].

The fact that inflammation disturbs the secretion of gonadotropins from the anterior pituitary (AP) in ewes was shown in our previous studies [12,13,14]. It is generally considered that immune/inflammatory challenge influences the secretion of LH via the suppression of GnRH secretion in the hypothalamus [13,15,16,17]. However, the antigonadotropic effect of inflammation could be more complex and may also involve the direct action of inflammatory mediators at the level of the pituitary gland. In the ex vivo study on the ovine AP explants, it was shown that pro-inflammatory cytokine interleukin (IL)-1β suppressed the secretion of LH [14]. This direct action of inflammatory mediators may occur due to the existence of their corresponding receptors in the AP [14,18,19]. Also, in our previous study it was revealed that mRNAs encoding pro-inflammatory cytokine receptors are expressed in the PT [20]. This suggests that the secretory activity of *PT* cells may be influenced by immune/inflammatory challenge.

Therefore, the recent study was designed to determine the role of melatonin in the modulation of LH secretion from the PT explants collected from saline- and endotoxin-treated ewes in the follicular phase of the oestrous cycle.

## 2. Results

### 2.1. Influence of GnRH and Melatonin on LH Releasing

**Figure 1:**
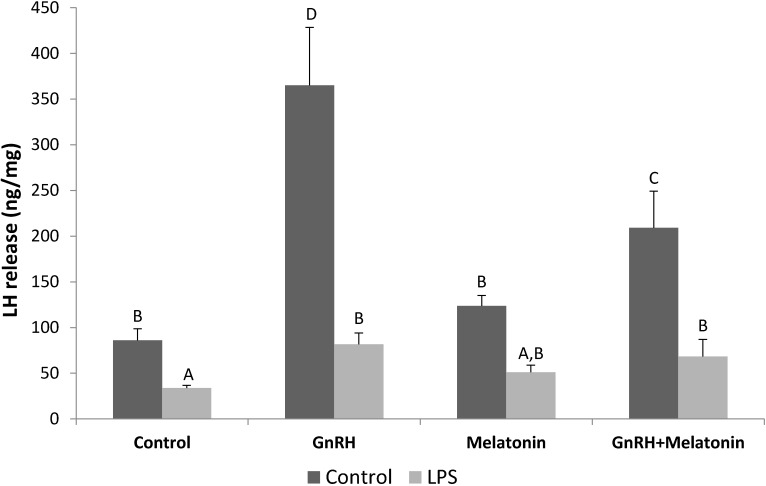
The effects of GnRH (100 pg/mL) and melatonin (10 nmol/mL) on LH release from the *Pars Tuberalis* explants. All data are presented as the mean (±S.E.M.). Different capital letters indicate significant (*p* < 0.05) differences according to a repeated-measures two-way analysis of variance (ANOVA, GraphPad Prism, San Diego, CA, USA) followed by a post hoc Sidak’s multiple comparison test.

LH release from the *PT* explants was stimulated (*p* < 0.05) by the GnRH treatment. Melatonin treatments reduced (*p* < 0.05) the stimulatory effect of GnRH on LH release only in explants collected from saline-treated ewes. On the other hand, no effect of melatonin treatment on the GnRH-induced release of LH was found in the *PTs* from LPS-injected animals. It is worth mentioning that explants collected from LPS-treated ewes were characterized by a lower (*p* < 0.05) GnRH-dependent response in LH release (Figure 1).

### 2.2. Influence of GnRH and Melatonin on LH Gene Expression

The treatment with GnRH stimulated (*p* < 0.05) the gene expression of LH in the *PT* explants from the saline-treated animals (Table 1). In the explants incubated separately with melatonin, no changes in the LH gene expression were found. However, in the explants co-incubated with GnRH, melatonin completely suppressed (*p* < 0.05) the stimulatory effect of GnRH on LH gene expression.

No effect of GnRH and melatonin on LH gene expression was found in the *PTs* from LPS-treated animals (Table 1).

### 2.3. Effect of GnRH and Melatonin on the Gene Expression of GnRHR, MT1, and Pro-Inflammatory Cytokines

No effect of GnRH and melatonin treatment on the gene expression of the GnRH receptor (GnRHR) in the *PT* explants was found. On the other hand, both GnRH and melatonin reduced (*p* < 0.05) MT1 mRNA expression in the *PTs* from saline-treated animals. Moreover, explants collected from LPS-treated ewes were characterized by the lower (*p* < 0.05) gene expression of GnRH and MT1 (Table 1).

Melatonin suppressed (*p* < 0.05) IL-1β, IL-6, and tumor necrosis factor (TNF) mRNA expression, but only in the *PT* explants collected from saline-treated ewes. Explants collected from endotoxin-treated animals were generally characterized by increased (*p* < 0.05) IL-6 mRNA expression but reduced (*p* < 0.05) TNF gene expression compared with the *PTs* collected from saline-treated ewes (Table 1).

## 3. Discussion

In the experiment it was shown that melatonin reduced GnRH-induced LH release in the ovine *PT* explants. This result confirms the previously published data showing that melatonin may be involved in the suppression of LH release from ovine *PTs* [21]. Moreover, the modulatory action of melatonin on LH secretion was also determined in rodents—in male rats, melatonin inhibited GnRH-induced LH release from the *PT*-ME explants [22]. This action of melatonin was mediated by the specific high-affinity membrane-bound receptors occurring in the *PT*. It was found that the intracellular mechanism of melatonin action is connected with a decrease in the intracellular calcium [Ca^2+^]_i_ level in the gonadotrophs because melatonin inhibits GnRH-induced Ca^2+^ release from the endoplasmic reticulum as well as Ca^2+^ influx through voltage-sensitive channels. Moreover, melatonin may decrease the influx of Ca^2+^ inhibiting GnRH-induced accumulation of cAMP, which, acting through protein kinase A, stimulates Ca^2+^ influx into the gonadotrophs [23,24]. Although the melatonin reduced GnRH-stimulated LH release in the present study, it did not influence the secretion of LH in the explants not treated with GnRH. The lack of melatonin effect on LH synthesis in the *PT* in a basal condition confirmed the previously published results of the ex vivo experiments on baboon primary pituitary cells [25]. The influence of melatonin on the LH secretion in ewes had also been studied in the in vivo experiments. In ovariectomized ewes with a melatonin implant, melatonin did not affect the serum level of LH. However, the stimulatory effect of GnRH intravenous injection on LH release was weaker in the animals with melatonin implants than in control animals [26]. The melatonin effect on LH secretion from the *PT* seems to be not connected with the changes in the tissue sensitivity to GnRH, since in our study it was shown that melatonin does not influence the gene expression of the GnRH receptor. Therefore, this is the first study to show the influence of melatonin on GnRHR expression in sheep. However, the suppressory effect of melatonin on the GnRHR expression in the European sea bass [27] may indicate that the effect of melatonin on the expression of GnRHR is species-dependent. In humans, melatonin treatment enhances the human chorionic gonadotropin (hCG)-stimulated progesterone secretion with inhibition of GnRH and GnRH receptor expression. In support of this, melatonin reduces both GnRH-I and GnRHR-I mRNA levels in human primary granulosa-luteal cells in a dose-dependent manner [28].

According to the results of the present study, it could be suggested that the physiological status of an animal may influence the responsiveness of the *PT* to both GnRH and melatonin action. These findings confirm the results of previous studies showing that the glands kept their ‘memory’ of the events triggered by LPS exposure, and this affected tissue explant activity during in vitro culture [14,29,30,31]. It was found that the *PT* explants isolated from ewes in an acute inflammatory state were characterized by significantly lower GnRH-dependent release of LH in comparison with the tissues collected from saline-treated individuals. Moreover, there was no effect of GnRH treatment on the gene expression of LH in the explants from LPS-treated animals. However, in the ex vivo study on the AP explants it was shown that GnRH stimulates the gene expression and release of LH regardless of the immune status of tissue-donor animals [14]. This suggests that inflammation may more strongly impair the secretory activity of the *PT* than the AP. The increased sensitivity of the *PT* cells to the inflammatory condition may result from the occurrence of a number of pro-inflammatory cytokine receptors with relatively high transcription, which was found in our previous study [20]. Moreover, our study showed that the secretory activity of the *PT* explants may be modulated by the locally synthesized pro-inflammatory cytokines, by which transcription was found in the explants even a few hours after the disappearance of the inflammatory stimuli. The *PT* explants collected from endotoxin-treated animals were characterized by higher transcription of IL-6, whereas the gene expression of TNF in those tissues was reduced. This suggests that the changes in secretory activity of the *PTs* collected from LPS-treated animals may be at least partially caused by the paracrine action of IL-6. Although the study on rats suggests the stimulatory effect of IL-6 on the release of LH [32], it could be assumed that IL-6 influences LH secretion via an indirect mechanism involving the antigonadotropic action of prolactin. It was previously found that IL-6 is a potent stimulator of prolactin secretion in the pituitary [33] which, in turn, may inhibit the secretion of LH in the paracrine manner [34]. Moreover, in the present study it was shown that the *PT* explants from the endotoxin-injected animals were not sensitive to melatonin action. This may have a profound importance to the circadian rhythm of changes in the secretion of LH. The induction of the *PT* insensitivity to melatonin action may be one of the mechanisms via which immune/inflammatory challenges disturb the reproduction process in animals.

In our study, it is suggested that the lower response of the *PT* explants collected from endotoxin-treated ewes to GnRH, and their insensitivity to melatonin action, may result from the reduced expression of GnRHR and MT1 in these tissues in comparison with explants from saline-treated animals. The reduced expression of GnRHR may directly influence LH release from the *PT* because the amount of GnRHR is considered a key factor determining the ability and storage strength of the pituitary gonadotropes in response to GnRH [35]. The fact that immune stress suppresses the pituitary sensitivity to GnRH stimulation [36] and the expression of GnRHR [13,15,16] has been previously confirmed in ewes. It is considered that the GnRHR expression decrease in the pituitary during an immune/inflammatory challenge results primarily from lower secretion of the hypothalamic GnRH [13,15,16,17], which is one of the most important regulators of its own receptor expression. GnRH activates the transcriptional activity of its own receptor gene through multiple pathways, including cAMP-, PKC-, and Ca^2+^-dependent signal transduction pathways [37]. However, the reduction of GnRHR gene expression during inflammation in the *PT* may also result from the direct action of pro-inflammatory cytokines and stress, because both IL-1β and corticotropin-releasing hormone (CRH) were shown to suppress GnRHR expression [14,38,39]. Moreover, the lowered GnRHR expression in the pituitary cells which is observed in the inflammatory response may result also from the direct action of the bacterial endotoxin, as toll-like receptor (TLR) 4 recognizing LPS is present in these cells [28,40,41]. It was previously reported that treatment with LPS decreased GnRHR gene expression as well as GnRH-induced secretion of LH in ovine AP explants [29].

Similarly, the melatonin insensitivity of *PT* explants collected from animals treated with LPS may result from decreased expression of MT1, which probably makes the *PT* cells unable to receive the melatonin signal. The membrane receptors MT1 and MT2 are considered to be the primary molecules mediating the receptor-dependent pathways of melatonin. The MT1 and MT2 belong to the class of G-protein-coupled receptors that, upon activation, trigger several signaling pathways, including the CREB, PI3K, and MAP kinase pathways [42]. The possible mechanism via which immune stress affects the expression of melatonin receptors in the *PT* and the effect of such influence have not been studied yet. The in vitro study suggests that the effect of inflammatory stimuli may be cell- and tissue-dependent as LPS treatment increased the number of melatonin binding sites in human monocyte cell cultures [43]. However, the decrease in the gene expression of MT1 in the *PT* may result from the stress induced by an immune/inflammatory challenge since stress decreases the density of melatonin binding sites in the suprachiasmatic nuclei [44]. In our study, it was also shown that both GnRH and melatonin treatments reduce the gene expression of MT1 in the *PT* explants from saline-treated animals. The suppressory effect of GnRH on MT1 expression has been previously evidently reported in an in vitro study on αT3-1 gonadotroph cell line [45]. The role of GnRH in the control of MT1 expression was also shown in an in vivo study on mice in which GnRH-deficient animals were characterized by elevated expression of MT1 in comparison with the control animals [46]. The inhibitory effect of melatonin on the gene expression of MT1 in the ovine *PT* explants fully supports the previous detailed studies in which the expression of MT1 mRNA was analyzed. It was found that the gene expression of MT1 in the *PT* undergoes circadian changes and melatonin is a strong suppressor of its expression [47].

It was previously described that the signaling cascade activated by LPS causes the translocation of the transcription factor—named the nuclear factor κ-light-chain-enhancer—of activated B cells (NF-κB), which binds to the promoters of target genes of proteins that mediate the innate immune response. At the beginning, proteins related to the pro-inflammatory phase are synthesized, such as pro-inflammatory cytokines, adhesion molecules, and enzymes (inducible nitric oxide (iNOS); cyclooxygenase (COX-2)). Secondary to this, the genes mediating the anti-inflammatory phase—such as the gene that codes for the inhibitory κB proteins—are activated. It is postulated that melatonin inhibits NF-κB activation and plays a direct role in ending the immune process [48]. These anti-inflammatory properties of melatonin have been previously found in both in vivo and ex vivo studies. Studies on aging rats showed that melatonin administration was able to abrogate changes on liver apoptosis induced by aging [49]. Ex vivo studies confirmed that, in LPS-stimulated macrophages, melatonin can suppress both iNOS and COX-2 expression by the inhibition of the p52 NF-κB-binding ability [50]. The study on rats showed that melatonin reduces mitochondrial oxidative damage and suppresses the IL-6 plasma level after venous infusion of LPS [51]. Moreover, melatonin was shown to attenuate neuroinflammation in rats. The study also showed that melatonin reduces the synthesis of pro-inflammatory cytokines and oxidative stress in the brain of rats that undergo intracerebroventricular administration of LPS [52]. Our results suggest that decreased MT1 gene expression may profoundly reduce the anti-inflammatory effect of melatonin on the *PT*. The study showed that melatonin influenced the gene expression of pro-inflammatory cytokines only in the explants collected from saline-treated animals, which were characterized by higher gene expression of MT1, but failed to influence the transcription of these cytokines in the tissues collected from LPS-treated individuals.

To summarize, in our ex vivo study it was shown that melatonin regulates the secretion of LH acting directly at the level of the *PT*. However, the immune status of the animal determines the sensitivity of the *PT* to this hormone action. This suggests that the inflammatory-dependent diminishing of the melatonin action on the pituitary may be one of the mechanisms via which immune/inflammatory challenges disturb the reproduction process in animals.

## 4. Materials and Methods

All procedures on animals were performed with the consent of the Local Ethics Committee of Warsaw University of Life Sciences–SGGW (Warsaw, Poland; authorization no. 50/2013).

The ex vivo experiments were carried out on the tissues collected from Blackhead ewes in the follicular phase of the oestrous cycle during the short-day photoperiod. For standardization of the experimental conditions, the oestrous cycles of the ewes were synchronized by the Chronogest^®^ CR (Merck Animal Health, Boxmeer, The Netherlands) according to the method described elsewhere [15].

The concentration of melatonin (Sigma-Aldrich, St. Louis, MO, USA) used in this study was chosen based on the preliminary experiment where the ability of melatonin concentrations in the range from 100 nM to 10 μM to modulate the release of LH from the *PT* explants was tested. The *PTs* were collected from five ewes and divided into five fragments each. The ex vivo incubation of the explants was performed in Medium 199 HEPES Modification (‘pure’ medium 199; Sigma-Aldrich, St. Louis, MO, USA) suitable for cell culture, with a penicillin–streptomycin solution at the dose of 10 mL/L (Sigma-Aldrich, St. Louis, MO, USA), and incubated at 37 °C, 87% O_2_, and 5% CO_2_. All the tissues were preincubated for 1 h in 800 μL of ‘pure’ medium 199. The explants were then divided into 5 groups: I, incubated with ‘pure’ medium 199; II, incubated with GnRH (100 pg/mL); III, treated with GnRH (Sigma-Aldrich, St. Louis, MO, USA) and melatonin (100 pmoL/mL); IV, treated with GnRH and melatonin (1 nmol/mL); and V, treated with GnRH and melatonin (10 nmol/mL). After 3 h of incubation, tissues and media were frozen at −80 °C until further assays. In the experimental media, the concentration of LH was determined by radioimmunoassay (RIA). The preliminary experiment showed that melatonin reduced the GnRH-induced LH release (260 ± 17 ng/mg) only at the dose of 10 μM (145 ± 25 ng/mg). Lower doses of melatonin did not significantly influence the concentration of LH in the experimental media. It is worth mentioning that the concentration of GnRH used in our study was chosen based on our previous study performed on the anterior pituitary explants [14].

In the main experiment, the animals were divided into two subgroups: control (*n* = 6) and LPS-treated (*n* = 6). Ewes were injected into the jugular vein with an appropriate volume of LPS from *Escherichia coli* 055:B5 (400 ng/kg) (Sigma-Aldrich, St Louis, MO, USA) dissolved in saline (0.9% *w*/*v* NaCl) (Baxter, Deerfield, IL, USA). The maximum volume of injected LPS solution (10 mg/L) never exceeded 2.5 mL. The control group received the same volume of NaCl (based on their body weight). Three hours after the LPS or saline injection, all animals had the rectal body temperature measured; the measurement results were 38.0 ± 0.3 °C in the saline-treated group and 39.6 ± 0.4 °C in the LPS-treated group. Three hours after the LPS or saline injection, all animals were euthanized by decapitation and the collected *PTs* were divided into 4 explants. The ex vivo incubation of the explants was performed analogously to the pilot experiment, in Medium 199 HEPES Modification (Sigma-Aldrich, St. Louis, MO, USA) with penicillin–streptomycin solution at the dose of 10 mL/L, (Sigma-Aldrich, St. Louis, MO, USA) and incubated at 37 °C, 87% O_2_, and 5% CO_2_. All the tissues were preincubated for 1 h in 800 μL of ‘pure’ medium 199. Then, the explants were divided into 4 experimental groups: I, incubated with ‘pure’ medium 199; II, treated with GnRH (100 pg/mL); III, treated with melatonin (10 nmol/mL); and IV, incubated with GnRH and melatonin. After 3 h of incubation, all explants and media were frozen at −80 °C until further assays.

### 4.1. Determination of the Relative Gene Expression

A NucleoSpin^®^ RNA kit (MACHEREY-NAGEL GmbH and Co, Düren, Germany) was used to isolate the total RNA from the explants. The purity and concentration of the isolated RNA was quantified spectrophotometrically with the use of a NanoDrop 1000 instrument (Thermo Fisher Scientific Inc., Waltham, MA, USA). The integrity of the isolated RNA was confirmed by electrophoresis with the use of 1% agarose gel stained with ethidium bromide. The Maxima™ First Strand cDNA Synthesis Kit for RT-qPCR (Thermo Fisher Scientific, Waltham, MA, USA) was used to perform cDNA synthesis. As a starting material for cDNA reversed transcription reaction synthesis, 2 μg of total RNA was used. Real-Time PCR was carried out with the use of the HOT FIREPol EvaGreen^®^ qPCR Mix Plus (Solis BioDyne, Tartu, Estonia) and HPLC-grade oligonucleotide primers (Genomed, Warszawa, Poland) according to the protocol described elsewhere [20]. The primer sequences were designed using Primer 3 software (Table 2). The relative gene expression was calculated using the comparative quantification option [53] of Rotor Gene 6000 software 1.7. (Qiagen, Dusseldorf, Germany). Three housekeeping genes were examined: glyceraldehyde-3-phosphate dehydrogenase (GAPDH), β-actin (ACTB), and cyclophilin C (PPIC). Because only a trace amount of mRNA encoding MT2 was detected in the *PT* explants, reliable analysis of the gene expression of MT2 was impossible to perform. The mean expression of these three housekeeping genes was used to normalize the expression of the analyzed genes. The results are presented in arbitrary units, as the ratio of the target gene expression to the mean expression of the housekeeping genes. The average relative quantity of gene expression in the control group of the *PT* explants collected from saline-treated ewes was set to 1.0.

### 4.2. Radioimmunoassay for LH

The concentration of LH in medium was assayed by double-antibody RIA using anti-ovine LH and anti-rabbit-c-globulin antisera and ovine standard (NIH-LHSO18), according to the previous report [55]. The assay sensitivity was 0.3 ng/mL and intra- and inter-assay coefficients of variation were 8.3% and 12.5%, respectively.

### 4.3. Statistical Analysis

The raw data, after passing the normality test, were subjected to a repeated-measures two-way analysis of variance (ANOVA, GraphPad Prism, San Diego, CA, USA) followed by a post hoc Sidak’s multiple comparison test. Statistical significance was established at *p* < 0.05. Data are presented as normalized to the control of saline-treated group.

## Figures and Tables

**Table 1:** The effects of GnRH (100 pg/mL) and melatonin (10 nmol/mL) on the gene expression of LH, GnRHR and MT1 in the *Pars Tuberalis* explants.

Gene	Animals	Group of *Pars Tuberalis* Explants			
		Control	GnRH	Melatonin	GnRH + Melatonin
LHβ	Saline-treated	1.00 ± 0.09 ^A^	1.57 ± 0.28 ^B^	1.04 ± 0.14 ^A^	0.74 ±0.10 ^A^
	LPS-treated	1.44 ± 0.18 ^A,B^	1.40 ± 0.22 ^A,B^	1.18 ± 0.27 ^A,B^	1.29 ± 0.19 ^A,B^
GnRHR	Saline-treated	1.00 ± 0.11 ^B^	1.36 ± 0.20 ^B^	1.28 ± 0.10 ^B^	1.40 ± 0.21 ^B^
	LPS-treated	0.56 ± 0.06 ^A^	0.77 ± 0.11 ^A^	0.72 ± 0.06 ^A^	0.79 ± 0.12 ^A^
MT1	Saline-treated	1.00 ±0.40 ^C^	0.50 ± 0.14 ^B^	0.38 ± 0.06 ^B^	0.36 ± 0.07 ^B^
	LPS-treated	0.18 ± 0.02 ^A^	0.25 ± 0.05 ^A^	0.46 ± 0.20 ^A,B^	0.18 ± 0.02 ^A^
IL-1β	Saline-treated	1.00 ± 0.08 ^D^	0.99 ± 0.04 ^D^	0.52 ± 0.06 ^A^	0.92 ± 0.14 ^B,C,D^
	LPS-treates	0.85 ± 0.06 ^B,C,D^	0.68 ± 0.08 ^B,C,D^	0.91 ± 0.07 ^C,D^	0.65 ± 0.07 ^A,B^
IL-6	Saline-treated	1 ± 0.06 ^B^	0.78 ± 0.12 ^A,B^	0.49 ± 0.04 ^A^	0.64 ± 0.06 ^A,B^
	LPS-treates	1.87 ± 0.22 ^C,D^	1.83 ± 0.21 ^C,D^	2.21 ± 0.27 ^D^	1.39 ± 0.23 ^B,C^
TNFα	Saline-treated	1 ± 0.06 ^C^	0.92 ± 0.07 ^B,C^	0.65 ± 0.10 ^A^	0.89 ± 0.09 ^A,B,C^
	LPS-treates	0.75 ± 0.07 ^A,B^	0.75 ± 0.08 ^A,B^	0.91 ± 0.07 ^B,C^	0.74 ± 0.10 ^A,B^

All data are presented as the mean (±S.E.M.). Different capital letters indicate significant (*p* < 0.05) differences according to repeated-measures two-way analysis of variance (ANOVA, GraphPad Prism, San Diego, CA, USA) followed by a post hoc Sidak’s multiple comparison test. Gene expression data were presented as normalized to the control of saline-treated group.

**Table 2:** All genes analyzed by Real-Time PCR are listed with their full names and abbreviations.

GenBank Acc. No.	Gene	Amplicon Size [bp]	Forward/Reverse	Sequence 5’→3’	Reference
NM_001034034	*GAPDH*	134	forward	AGAAGGCTGGGGCTCACT	[16]
	glyceraldehyde-3-phosphate dehydrogenase		reverse	GGCATTGCTGACAATCTTGA	
U39357	*ACTB*	168	forward	CTTCCTTCCTGGGCATGG	[16]
	beta actin		reverse	GGGCAGTGATCTCTTTCTGC	
NM_001076910	*PPIC*	131	forward	ACGGCCAAGGTCTTCTTTG	[16]
	cyclophilin C		reverse	TATCCTTTCTCTCCCGTTGC	
X52488	*LHβ*	184	forward	AGATGCTCCAGGGACTGCT	[16]
	luteinizing hormone beta-subunit		reverse	TGCTTCATGCTGAGGCAGTA	
NM-001009397	*GnRHR*	150	forward	TCTTTGCTGGACCACAGTTAT	[16]
	gonadotropin-releasing hormone receptor		reverse	GGCAGCTGAAGGTGAAAAAG	
U02517	*GnRH*	123	forward	GCCCTGGAGGAAAGAGAAAT	[16]
	gonadotropin-releasing hormone		reverse	GAGGAGAATGGGACTGGTGA	
XM_614283.6	*MT1*	114	forward	GCCTCCATCCTCATCTTCAC	Originally designed
	Melatotonin receptor type I		reverse	GCTCACCACAAACACATTCC	
NM_001130938	*MT2*	107	forward	CTCCGGAACGCAGGTAAC	Originally designed
	Melatotonin receptor type II		reverse	CAGCCGTCGTGGAAGATG	
X54796.1	***IL1B***	137	forward	CAGCCGTGCAGTCAGTAAAA	[54]
	interleukin 1 beta		reverse	GAAGCTCATGCAGAACACCA	
NM_001009392.1	***IL6***	165	forward	GTTCAATCAGGCGATTTGCT	[54]
	interleukin 6		reverse	CCTGCGATCTTTTCCTTCAG	
NM_001024860	***TNF***	153	forward	CAAATAACAAGCCGGTAGCC	[54]
	tumor necrosis factor		reverse	AGATGAGGTAAAGCCCGTCA	
